# Comparison of machine learning methods for estimating case fatality ratios: An Ebola outbreak simulation study

**DOI:** 10.1371/journal.pone.0257005

**Published:** 2021-09-15

**Authors:** Alpha Forna, Ilaria Dorigatti, Pierre Nouvellet, Christl A. Donnelly

**Affiliations:** 1 School of Computing Science, Simon Fraser University, Burnaby, British Columbia, Canada; 2 Department of Infectious Disease Epidemiology, MRC Centre for Global Infectious Disease Analysis, Imperial College London, London, United Kingdom; 3 School of Life Sciences, University of Sussex, Brighton, United Kingdom; 4 Department of Statistics, University of Oxford, Oxford, United Kingdom; Taipei Medical University, TAIWAN

## Abstract

**Background:**

Machine learning (ML) algorithms are now increasingly used in infectious disease epidemiology. Epidemiologists should understand how ML algorithms behave within the context of outbreak data where missingness of data is almost ubiquitous.

**Methods:**

Using simulated data, we use a ML algorithmic framework to evaluate data imputation performance and the resulting case fatality ratio (CFR) estimates, focusing on the scale and type of data missingness (i.e., missing completely at random—MCAR, missing at random—MAR, or missing not at random—MNAR).

**Results:**

Across ML methods, dataset sizes and proportions of training data used, the area under the receiver operating characteristic curve decreased by 7% (median, range: 1%–16%) when missingness was increased from 10% to 40%. Overall reduction in CFR bias for MAR across methods, proportion of missingness, outbreak size and proportion of training data was 0.5% (median, range: 0%–11%).

**Conclusion:**

ML methods could reduce bias and increase the precision in CFR estimates at low levels of missingness. However, no method is robust to high percentages of missingness. Thus, a datacentric approach is recommended in outbreak settings—patient survival outcome data should be prioritised for collection and random-sample follow-ups should be implemented to ascertain missing outcomes.

## Introduction

Machine learning (ML) algorithms—computer algorithmic techniques that learn patterns in data—are increasingly used in epidemiology [1, 2] in areas including diagnostics, predictive analytics, and missing data imputation [1–4]. Forna *et al*. used Boosted Regression Trees (BRTs), a ML approach, to impute missing survival outcomes, adjust imputations for model imperfection and combine the imputations with observed outcomes to re-examine case fatality ratio (CFR) estimates (i.e. the probability that cases die due to the infection) for the West African Ebola epidemic [5]. Those CFR estimates corroborate estimates from Garske *et al*. where an overall mean CFR of 62.9% (95% CI: 61.9% to 64.0%) was reported for confirmed cases with recorded clinical outcomes [6].

ML methods typically achieve encouraging out-of-sample performance levels, often with minimal data pre-processing [7]. For example, BRTs allow for missingness in predictors, capture complex nonlinear relationships between outcomes and predictors, and are insensitive to outliers within the predictors [8]. However, a major challenge is the need skilfully to optimise hyperparameters to maximise out-of-sample performance. Additionally, it is unclear how data characteristics, such as the type of missingness, influence the performance of ML algorithms and the estimates based, in part, on the imputed data.

Thus, we sought ML methods that are resilient to hyperparameter choices. However, the literature suggests that even when hyperparameters are minimally dependent on data, ML methods perform best when trained on data for the specific problem for which they are to be implemented [9, 10]. Beyond the need to optimise ML algorithms before they are fit for purpose [11], practical challenge in the use of ML algorithms for epidemiological analysis remains how they perform for various outbreak data characteristics, not least, the scale and type of missingness.

In this simulation study, we characterise the inferential and predictive performance of five algorithms (i.e., logistic regression (LR), random forest (RF), BRTs, Bayesian Additive Regression Trees (BART) and Artificial Neural Network (ANN)) to estimate CFR for Ebola virus disease (EVD) in the presence of missing survival outcome data. Specifically, we vary the simulated size of the outbreak dataset, the type and scale of data missingness, and the model training/validation ratio. Our aim was not to perform a technical appraisal of ML methods as in previous studies [1, 12], but rather to demonstrate how different ML methods behave under different (simulated) infectious disease outbreak data characteristics, focusing on the scale and type of survival outcome missingness.

## Methods

### Simulated data

We simulated an outbreak dataset of 12,049 cases (i.e. complete case count of confirmed, probable, suspected cases) to mimic that of the 2013–2016 Ebola epidemic in West Africa [13]. We included 20 predictors and the survival outcome variable. Predictors included demographic predictors such as age group, case classification and clinical predictors such as fever occurrence and unexplained bleeding (S1 Appendix). We let ***X*** = {X_1_, X_2_, …, X_20_} be a vector of the predictors sampled with replacement from the observed dataset such that the distribution of each simulated predictor is similar to that of its observed progenitor. The survival outcome variable was simulated such that 76.5% died and 23.5% survived, similar to the pattern observed in the West African Ebola epidemic dataset [5]. Using the coefficients from a generalised linear model, the probability of survival ***Y*** was modelled as a function of ten predictors (***X***^***s***^ ⊂ ***X***): age, country, reporting delay, case classification, hospitalisation status, quarter (date of reporting aggregated at 3-month intervals), difficulty breathing, fever, fatigue, and anorexia. The uniform distribution with bounds of 0 and 1 was used to generate the survival probabilities. The generalised linear model could be written as:

$$g(Y)=X^{s}\beta +\varepsilon$$

The parameter *ε* is the error distribution and β are coefficients of the model. β could be written as a 2×1 matrix as follows:

$$\beta =\left[\begin{array}{c} \beta_{o}\\ \beta_{k} \end{array}\right]$$

The parameter *β*_*o*_ is the coefficient of the intercept and *β*_*k*_ are the coefficients of the simulated predictors.

Thus, *β*^*T*^, the transpose of the 2×1 coefficient matrix for the simulated (i.e., generalised linear model) model is as follows:

$$\beta^{T}=\left[\begin{array}{cccccccccc} -0.13 & 0.11 & 0.25 & 0.31 & 0.21 & 0.2 & 0.13 & 0.16 & 0.11 & 0.13\\ 0.11 & 0.10 & 0.05 & 0.06 & -0.02 & 0.05 & -0.04 & -0.03 & 0.01 & -0.03\\ -0.11 & 0.21 & 0.20 & 0.20 & 0.22 & 0.19 & 0.21 & 0.13 & 0.09 & -0.05\\ -0.01 & -0.03 & & & & & & & & \end{array}\right]$$

In this matrix, for the categorical predictors, age has 16 levels, country and case classification have three levels each, quarter has seven levels, and all the other categorical predictors have 2 levels each. Reporting delay is a continuous predictor with mean = 5.68 days and standard deviation = 8.91 days (S1 Appendix).

To these predictors in the simulated model, we added ten others (***X***^***a***^ ⊂ ***X***): unexplained bleeding, confusion, joint pain, jaundice, conjunctivitis, vomiting, diarrhoea, headache, muscle pain and chest pain. These added predictors independent from the outcome, were used to fit the ML models.

### Algorithms evaluated in this study

The five algorithms (i.e., LR, RF, BRT, BART, and ANN) used for these experiments are briefly described in the supplementary information (S1 Appendix). With exception of LR, the algorithms all have tuneable hyperparameters. These tuneable hyperparameters and their purposes are summarised in Table 1.

**Table 1:** Summary of the machine learning algorithms and hyperparameters investigated.

ML algorithms	Hyperparameters tuned	Function of the hyperparameter in the model	Hyperparameter space considered (lower bound, upper bound)
Logistic Regression (LR)	–		–
Random Forest (RF) [14]	Ntree	Number of trees	(100,1000)
	Mtry	Number of predictors randomly selected as candidates for splitting a node	(3,10)
	Nodesize	Forest average number of unique data points in a terminal node	(10,30)
Boosted Regression Trees (BRTs) [15]	Ntrees	Integer specifying the total number of trees to fit	(100,2000)
	interaction.depth	Integer specifying the maximum depth of each tree (i.e., the highest level of predictor interactions allowed)	(2,10)
	bag.fraction	Fraction of training dataset observations randomly selected for each tree. This introduces random variation into the model fit	(0.5,0.75)
	shrinkage (Also known as the learning rate)	Shrinkage parameter applied to each tree	(0.001,0.05)
Bayesian Additive Regression Trees (BART) [16]	num_trees	The number of trees to be grown in the sum-of-trees model.	(5,20)
	num_burn_in	Number of MCMC samples to be discarded as “burn-in’’	(10,30)
	num_iterations_after_burn_in	Number of MCMC samples to draw from the posterior distribution of the fitted function	(100,300)
	Beta	Power hyperparameter in tree prior distribution for whether or not a node is nonterminal	(1,2)
Artificial Neural Network (ANN) [17]	Size	Number of units in the hidden layer	(5,20)
	Decay	Parameter for weight decay	(10^−8^,0.002)
	Abstol	Value below which the modelling fitting is stopped to prevent overfitting	(0.001,0.002)
	Reltol	If the optimizer is unable to reduce the fit criterion by a factor of at least (1–reltol), modelling fitting is stopped	(10^−8^,10^−5^)

We briefly describe the outbreak data characteristics investigated in this simulation study.

### Outbreak dataset size

Infectious disease outbreak sizes vary from one outbreak to another. In this simulation study, to investigate outbreak dataset size, we down sampled the simulated dataset from 100% of cases (12,049 cases) to 75% (9,037 cases), and 50% (6,025 cases) of cases and tested in each scenario for model performance and CFR estimation.

### Type and scale of missingness

Infectious disease outbreak datasets are almost never complete, and this data missingness can introduce biases in estimating epidemiological parameters (e.g., CFR) from outbreak data. Data missingness can be classified into three mechanisms: missing completely at random (MCAR), missing at random (MAR) and missing not at random (MNAR) [18] MCAR means that the data are missing non-systematically and that any sample drawn from the data is representative of the underlying population [18]. MAR means that the probability of missingness in a predictor is conditional on predictors (i.e., explanatory predictors) in the data. Finally, MNAR means that the probability of missingness in a predictor is conditional on the outcome (i.e., dependent variable). Details of how these different mechanisms were simulated are included in the supplementary information (S1 Appendix). For instance, we confirmed missingness was MNAR by ensuring that the missingness in survival outcome was conditional on the probability of the outcome itself. Our simulation study tests the robustness of the ML algorithms in estimating unbiased CFRs given the mechanism underlying the missingness.

To investigate the proportion of missingness, we generated 10%, 20% and 40% of each type of missingness in the survival outcome variable. For each proportion of outcome missingness, the same proportion of missingness in the predictors is simulated as MCAR (i.e., assuming that the missingness in all the predictors is MCAR).

### Model training/Validation ratio

The training/validation ratio (proportion of training data) has a direct influence on model hyperparameterisation [19]. In this simulation study, we tested three training/validation splits of the datasets: (i) 50% data for training and 50% for validation; (ii) 65% data for training and 35% for validation; and (iii) 80% data for training and 20% for validation.

### Experimental setup

We simulated the survival outcomes with ten predictors but fitted models with 20 potential predictors. To investigate whether the methods eliminate the ten irrelevant predictors from the simulation, we estimated how important individual predictors were by contrasting inferential performances. Inferential performance, measured as the mean squared error (mse), characterises the change in performance from permuting the values of each predictor and comparing those to predictions made on the unpermuted simulated data [20].

In fitting each method to the data, we performed 5-fold cross validation. In cross validation, the dataset is divided into *k* sub-samples (in our case *k* = 5). A single sub-sample is chosen as testing data, and the remaining *k* − 1 sub-samples are used as training data. The procedure is repeated *k* times, in which each of the *k* sub-samples is used exactly once as the testing data [21]. The *k* results are averaged, and the resulting single estimate is used to evaluate each method during hyperparameter optimisation.

For each method considered (LR, RF, BRT, BART and ANN), we used the literature to guide the choice of upper and lower bounds of the hyperparameter values used to optimise performance (Table 1) [8, 22–25], Conditional upon these bounds, 50 random grid searches were carried out to identify the optimised hyperparameters for each algorithm. These optimised hyperparameters were then used for model validation (using data held out for model validation) and CFR estimation based on data with simulated missingness (both predictors and outcome). For model validation, outcome imputation performance was characterised using the sensitivity, specificity, percentage correctly classified (PCC) and the area under the receiver operating characteristic curve (AUC). Each model returned a probability for each missing outcome. Converting these probabilities into imputed binary values required the identification of a threshold probability. We selected our threshold to obtain equal sensitivity and specificity [5].

Within this algorithmic framework, we varied the outbreak dataset size, the type and scale of data missingness, and the model training/validation ratio for 100 simulations, each time estimating the model performance and CFR. The true CFR of the simulated dataset was also calculated each time. A step-by-step detailed description of the algorithmic framework is provided in the supplementary information (S1 Appendix).

In earlier work, assuming survival outcomes were MAR, Forna *et al*. adjusted the imputed CFR to account for imperfect sensitivity and specificity of the BRT method [5]. As a sensitivity analysis, we adjusted the unadjusted CFR estimates to investigate whether adjusting the imputed survival outcomes reduces the bias in the true CFR for all methods. The reduction in bias was calculated as follows:

$$Unadjusted\;CFR\;bias=\left|Unadjusted\;CFR-True\;CFR\right|$$

$$Adjusted\;CFR\;bias=\left|Adjusted\;CFR-True\;CFR\right|$$

$$Reduction\;in\;bias\;(percent)=\frac{Adjusted\;CFR\;bias}{Undjusted\;CFR\;bias}\times 100,$$ (1)

The *mlr* package (version 2.19.0); which provides a unified interface to ML in R was utilised for fitting the models [26]. The *RSurveillance* package (version 0.2.1); which adjusts for model sensitivity and specificity was used for CFR estimation [27]. The ‘optimal.threshold’ function in the *PresenceAbsence* (version 1.1.9) package was used to achieve equal sensitivity and specificity for CFR prediction [28]. R (version 3.6.2) was used for all analyses.

Algorithms LR and ANN do not allow for missingness in predictors, therefore we imputed the missing predictor values using the mean observed predictor values to allow all algorithms to be applied to the same sized datasets.

The simulated outbreak dataset and the algorithmic pipelines used to investigate the different ML methods and how they behave under different (simulated) infectious disease outbreak data scenarios are available on GitHub: https://github.com/Paalpha/ebola_out_simulation.

## Results

**Fig 1:**
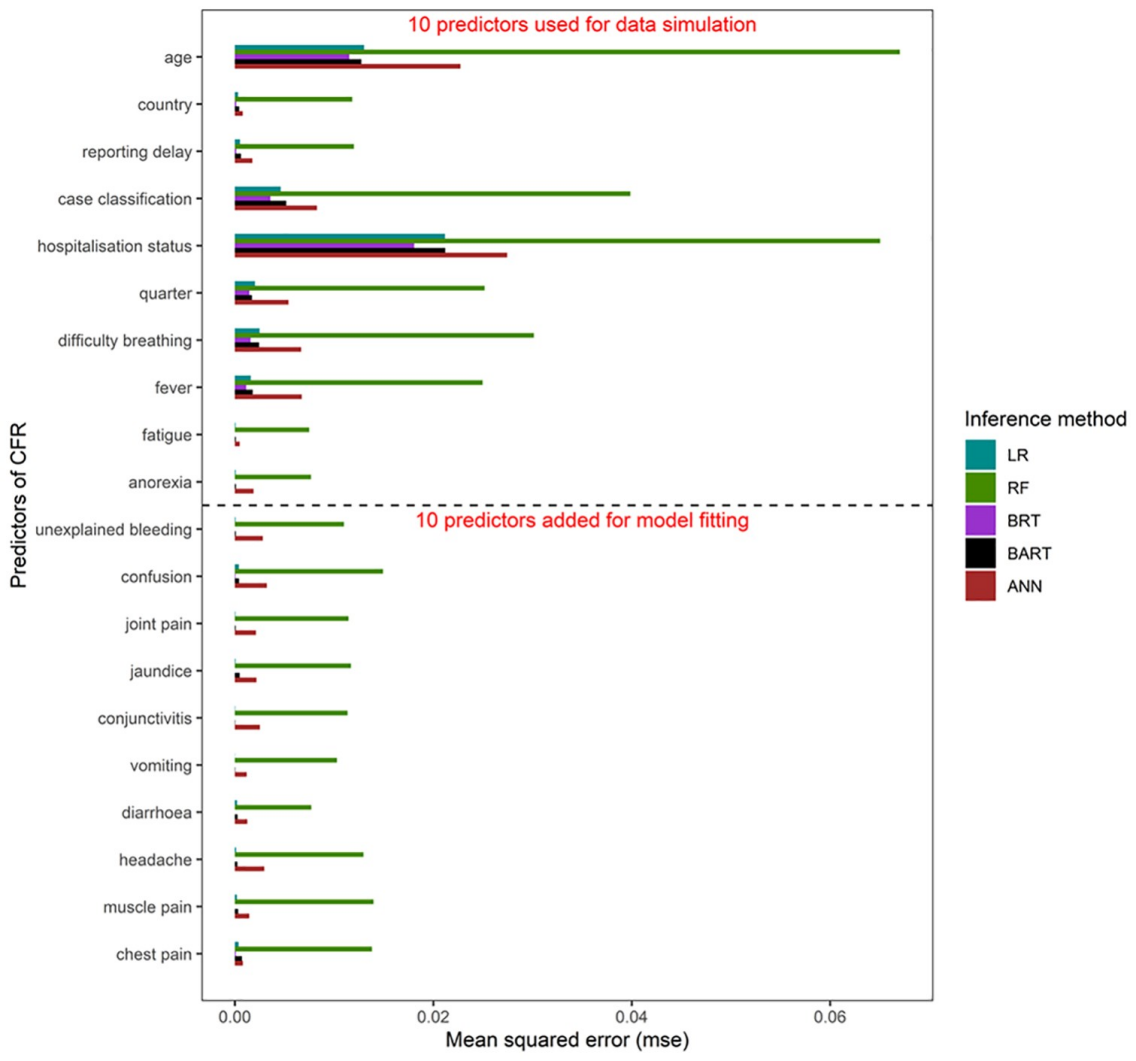
Inferential performances (mean squared error [mse]) for all 20 predictors of Case Fatality Ratio (CFR) estimated for each method (i.e., LR, RF, BRT, BART, ANN). The dotted horizontal line separates the ten predictors used in the simulation process from the ten predictors added for model fitting.

Fig 1 shows the inferential performances for all 20 predictors and each method. Across methods, the inferential performance (mse) for the ten predictors included in the simulation process was 1% (median, range: 0%–6.7%) higher than that for the other predictors added for model fitting 0.3% (median, range: 0%–1.5%).

**Fig 2:**
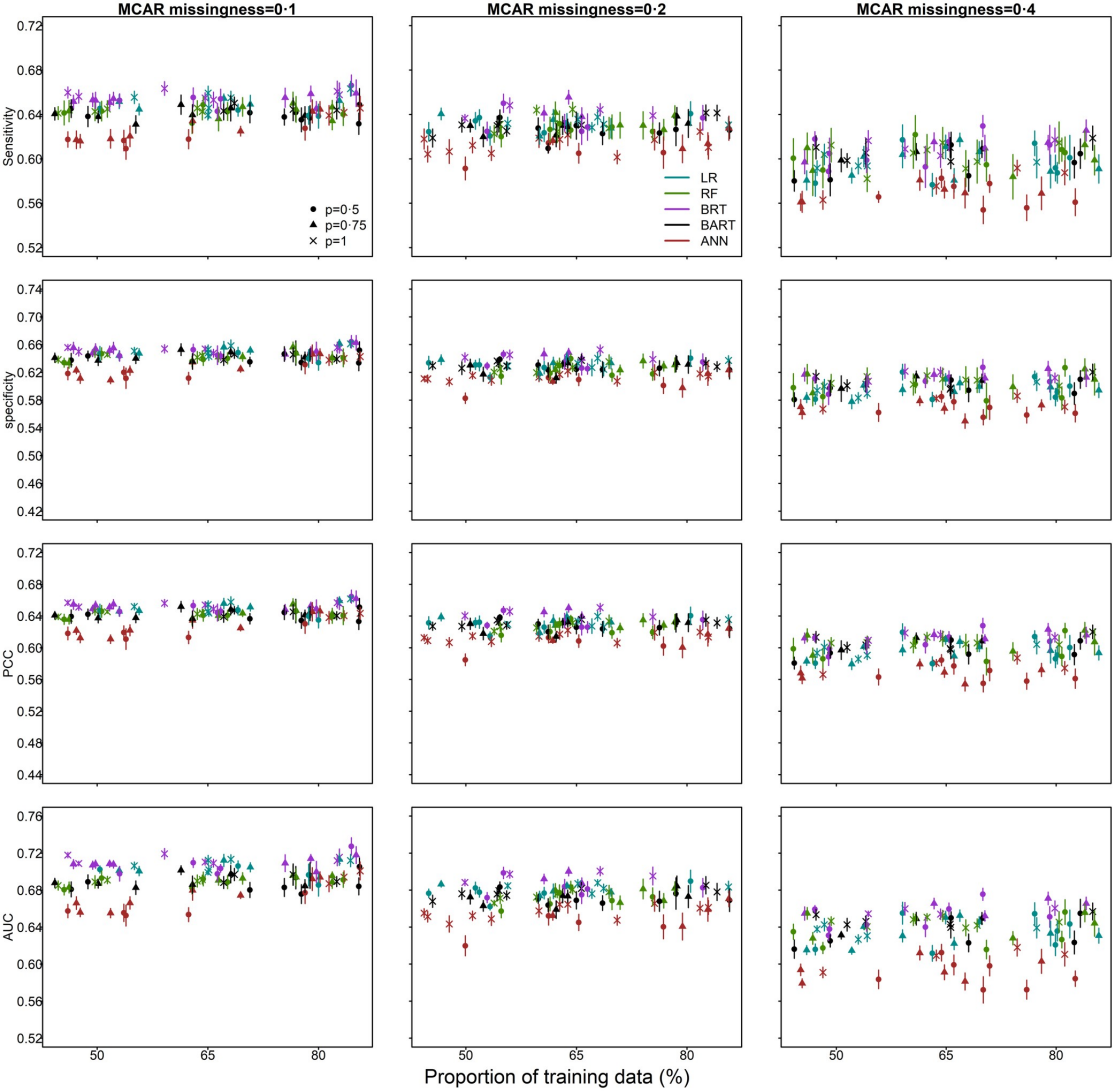
Survival outcomes and predictors missing completely at random (MCAR): Imputation performance (sensitivity, specificity, percentage correctly classified [PCC], area under the receiver operating characteristic curve [AUC]) as a function of the proportion of data used for model training and a) the proportion of outbreak data (*p*); where *p* = 1 corresponds to the full dataset of 12,049 cases (100%); b) the proportion of simulated missingness of 0.1 (10%), 0.2 (20%) and 0.4 (40%). Median and 95% confidence intervals are plotted. (The horizontal axis has been jittered for readability).

The performance of the models decreased as the data proportion of missingness increased. For instance, across methods, dataset size and proportion of training data used, the AUC decreased by 7% (median, range: 1%–16%) when missingness was increased from 10% to 40% (Fig 2). At 40% missingness, the AUC of BRTs was slightly greater, 2% (median, range: -14%–16%), compared to the other methods combined (Fig 2). Across methods, the proportion of missingness and proportion of training data used, the AUC increased by 1% (median, range: -4%–6%) when outbreak size was increased from 50% to 100%. The AUC increased by 1% (median, range: -5%–4%) when the proportion of data used for model training increased from 50% to 80% (Fig 2).

**Fig 3:**
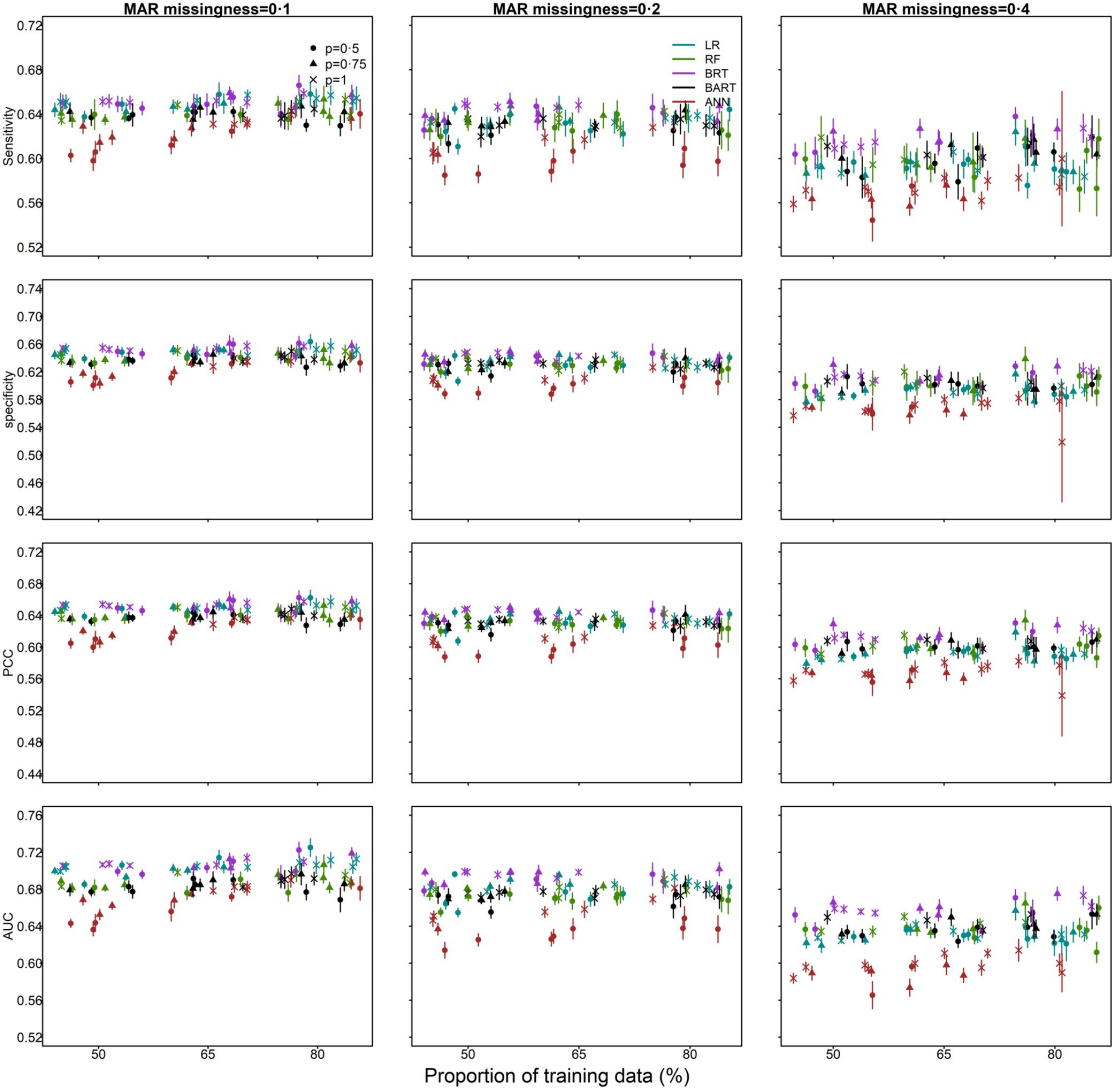
Survival outcome missing at random (MAR) and predictors missing completely at random (MCAR): Imputation performance (sensitivity, specificity, percentage correctly classified [PCC], area under the receiver operating characteristic curve [AUC]) as a function of the proportion of data used for model training and a) the proportion of outbreak data (*p*); where *p* = 1 corresponds to the full dataset of 12,049 cases (100%); b) the proportion of simulated missingness of 0.1 (10%), 0.2 (20%) and 0.4 (40%). Median and 95% confidence intervals are plotted. (The horizontal axis has been jittered for readability).

Fig 3 shows performance results for MAR missingness in the survival outcome and MCAR missingness in the predictors. The performance profile for this scenario is similar to that in the MCAR simulations.

**Fig 4:**
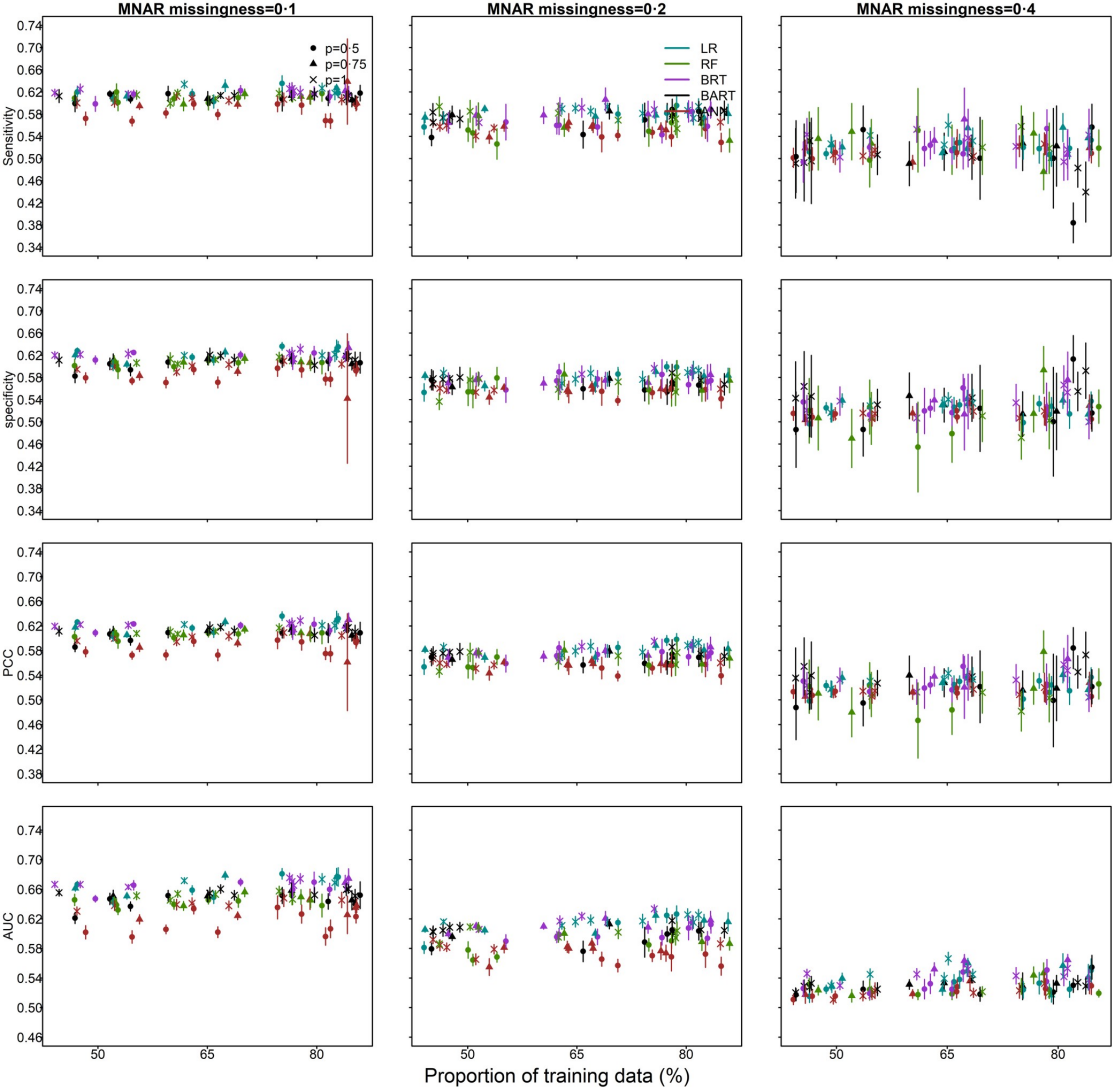
Survival outcome missing not at random (MNAR) and predictors missing completely at random (MCAR): Imputation performance (sensitivity, specificity, percentage correctly classified [PCC], area under the receiver operating characteristic curve [AUC]) as a function of the proportion of data used for model training and a) the proportion of outbreak data (*p*); where *p* = 1 corresponds to the full dataset of 12,049 cases (100%); b) the proportion of simulated missingness of 0.1 (10%), 0.2 (20%) and 0.4 (40%). Median and 95% confidence intervals are plotted. (The horizontal axis has been jittered for readability).

Below 20% missingness, methods performed consistently better than chance (AUC 62% (median, range: 53%–68%)), when selecting a probability threshold such that sensitivity equals specificity (Fig 4). AUC decreased by 12% (median, range: 6%–16%) when the missingness was increased from 10% to 40% (Fig 4). At 40% missingness, sensitivity, specificity and PCC and estimates overlapped for most methods, and AUC estimates were only slightly above 50%. Similar to MCAR, performance for MAR improved with increased outbreak dataset size and, on average, performance slightly improved with increases in the percentage of data used for model training.

**Fig 5:**
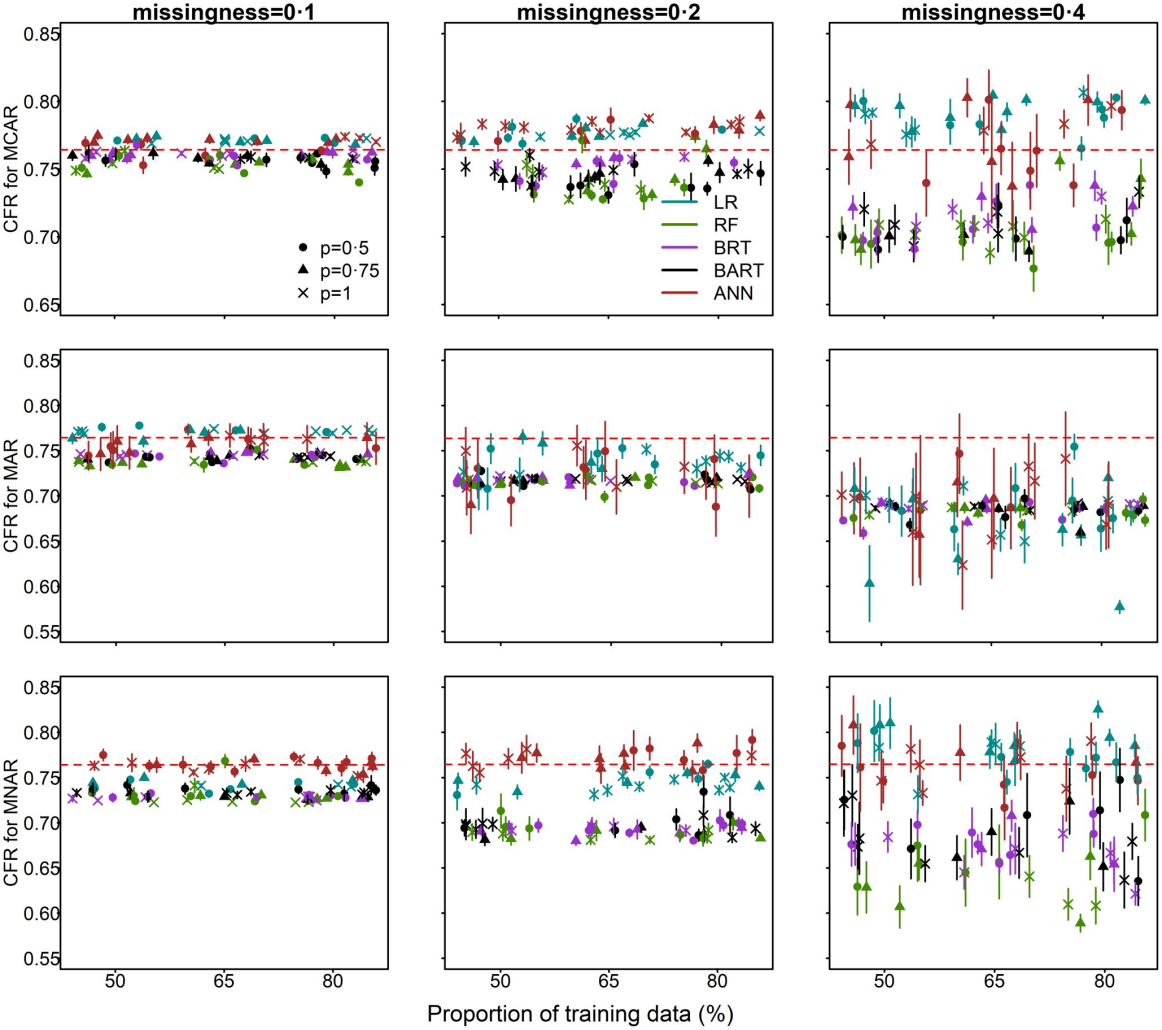
The relationship between the unadjusted CFR estimate for each missingness type (MCAR, MAR, MNAR) and the proportion of data used for model training and a) proportion of outbreak data (*p*); were *p* = 1 corresponds to the full dataset of 12,049 cases (100%); b) proportion of simulated missingness of 0.1 (10%), 0.2 (20%) and 0.4 (40%). The true CFR of the complete simulated data (without simulated missingness) is indicated by the red dotted horizontal line. Median and 95% confidence intervals are plotted. (The horizontal axis has been jittered for readability).

For all methods considered in these experiments, the bias in CFR increased with increasing proportions of missingness (Fig 5). For MCAR missingness, LR and ANN tended to overestimate the CFR while RF, BRT and BART tended to underestimate the CFR. For MAR missingness, all models tended to underestimate the CFR, with increasing uncertainty compared to the CFR estimates obtained with MCAR. For MNAR missingness, the bias and uncertainty in the CFR estimates were comparatively higher. There were no distinguishable patterns in CFR estimates due to differences in outbreak dataset size. The uncertainty in CFR estimates usually increases with outbreak size. However, in our simulation this was not observed. The proportion and type of missingness in survival outcomes are therefore driving the uncertainty in CFR. Finally, the CFR estimates appeared to be independent of the training/validation ratio.

**Fig 6:**
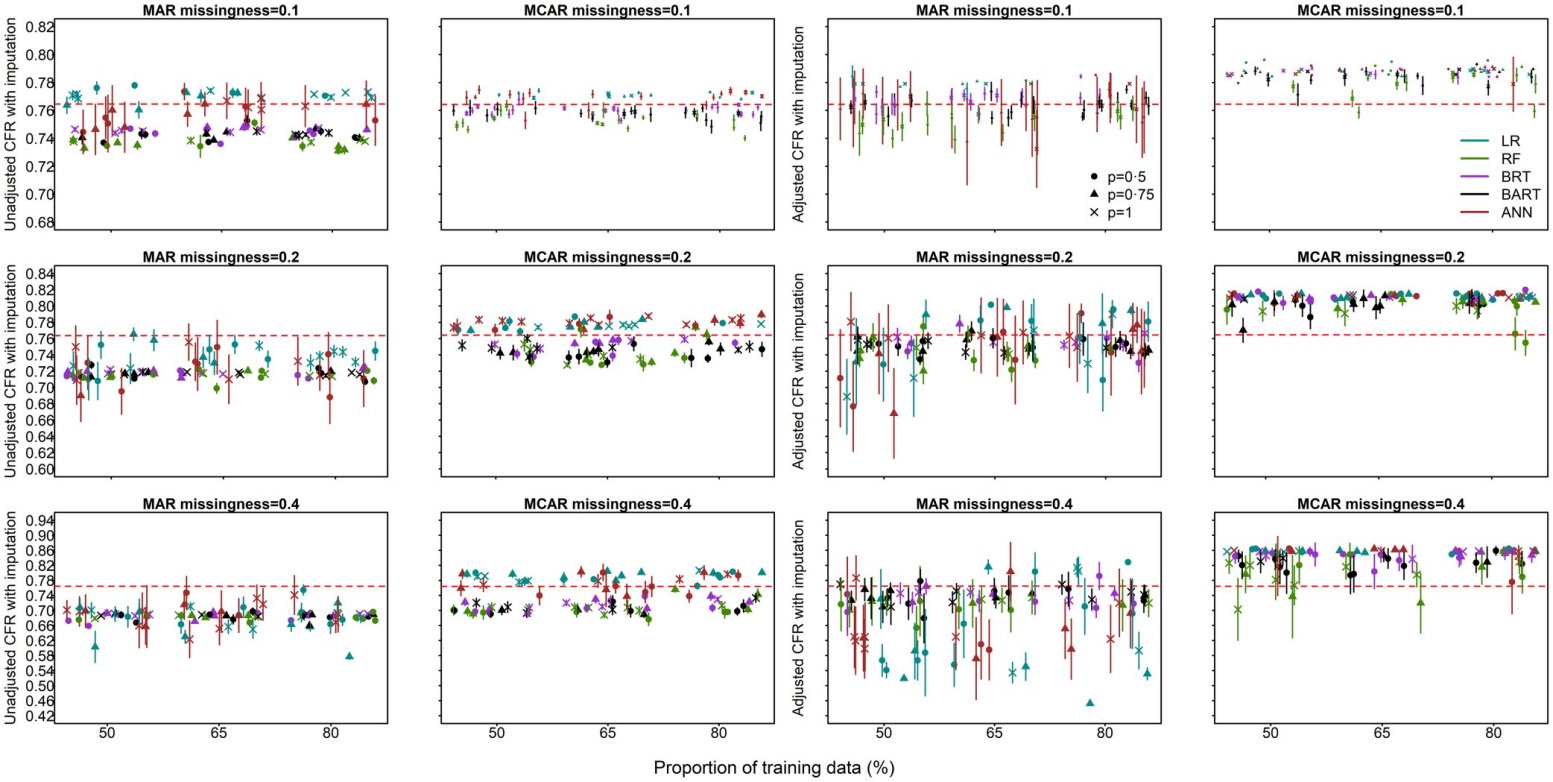
The unadjusted and adjusted CFR estimates for MAR and MCAR missingness in survival outcomes and predictors Missing Completely at Random (MCAR). The true CFR of the complete simulated data (without simulated missingness) is indicated by the red dotted horizontal line. Median and 95% confidence intervals are plotted. (The horizontal axis has been jittered for readability).

For MAR, the overall reduction in bias across methods, proportion of missingness, outbreak size and proportion of training data was 0.5% (median, range: 0%–11%), while the overall reduction in bias for MCAR was 2.25% (median, range: 0%–10%) (Fig 6). MCAR-adjusted CFRs are upwardly biased when missingness is 20% or 40% but the MAR-adjusted CFRs are relatively less biased for the same proportions of missingness (Fig 6).

## Discussion

ML algorithms are used in various epidemiological applications [29–31]. An increasingly common application is to obtain and improve estimates of epidemiological parameters of infectious diseases [32]. Our simulation study examined the inferential and predictive performance of five ML algorithms (i.e., LR, RF, BRT, BART and ANN) to estimate the CFR for Ebola varying outbreak data characteristics including the outbreak dataset size, the type and magnitude of data missingness, and the model training/validation ratio.

The inferential performance profile (Fig 1) shows that the methods correctly identified the ten predictors that largely explained the simulated variance in CFR. RF picks up signals from predictors not used in the simulation because it is inherently programmed to explore predictors independent of data during model fitting as a way of increasing model generalisability [19].

Corroborating the existing literature, our simulations demonstrate that ML algorithms perform better with large (outbreak) database sizes [33]. Algorithms like ANN usually require even more data to achieve optimal performance and produce unbiased CFR estimates. The suboptimal performance for outbreaks of small sizes can be attributed to less information in small datasets resulting in ML classifiers that are less generalisable. In practice, the amount of data is an important factor to consider when deciding whether to use ML to impute survival outcomes.

The type and scale of missingness in the survival outcomes substantially influence the performance of ML algorithms. The performance profile of the methods for MCAR and MAR missingness is consistent with the published literature [34–36]. For example, missing data handling methods like listwise deletion and multiple imputations have been previously shown to be unbiased in the presence of MCAR and MAR missingness [34]. In addition, consistent with published work [34], we found that as the proportion of missingness increased, none of the ML algorithms performed ideally for survival outcome MNAR missingness. In fact, no inferential method performed particularly well when >20% of outcomes are MNAR. Thus, MNAR CFR estimates showed more bias and more uncertainty. In instances of >20% missingness in survival outcomes, at least 50% random follow up of cases could reduce the bias and uncertainty in CFR estimates. For missingness MAR, CFR estimates were below the true CFR. Our simulations show that for missingness > 10% imputation using ML may bias CFR estimates, in line with existing literature suggesting that datasets with limited missingness are not influenced to the same extent by data imputation methods as compared to datasets with substantial levels of missingness [37]. Based on these results, epidemiologists should investigate the scale and type of missingness (using statistic tests such as Little’s MCAR test [38]) before using ML algorithms for CFR estimation. For instance, the scale of MNAR missingness would be greater in the early stages of an epidemic than later as follow-up methods, laboratory testing, reporting lag and case-identification improve. Thus, key domain knowledge and outbreak context should drive data analysis and interpretation of results.

We found that model performance increases with increases in model training/validation ratio. These results suggest that provided the outbreak dataset is sufficiently large, training ML models on at least half (50%) of the data could optimise performance as techniques like *k*-fold cross validation ensure that models are internally validated to prevent overfitting to training data.

Our results do not investigate the type of missingness in the predictors of survival outcome; we assumed that the missingness in all predictors was MCAR. We also assume that predictors are MCAR with the same percentage as the outcome missingness. Thus, these experiments are a simplification of the possible complexities in observed outbreak data. We investigated the proportion of missingness in the predictors and imputed the missing values using the mean before implementing LR and ANN. Imputing for missingness in predictors, at least in part, could explain the relatively reduced performance of these two algorithms. ANN parameters usually require expert optimisation which may explain part of the reduction in performance. RF, BRT and BART algorithms as implemented in our experiments inherently handled the missingness in the predictors [23, 24]. By resampling with replacement from the 2013–2016 West African Ebola outbreak dataset, these simulation results are only contextually relevant to an infinite population with the exact characteristics of that dataset.

This work is not a technical appraisal of ML methods, but rather it is meant to guide epidemiologists in making more informed choices as they consider the use of the available ML tools for CFR estimation and, more broadly, for infectious disease outbreak analysis.

The ML methods investigated in this paper are not exhaustive. Methods like XGBoost, a variant of tree ensembled models have shown high performances in previous experiments [39]. Automated machine learning methods like Tree-based Pipeline Optimization Tool (TPOT) that do data cleaning, predictor engineering, model selection, and hyperparameter optimisation in one operation are rapidly been developed and deployed [40]. While these advanced approaches would ultimately make ML more accessible to epidemiologists, misleading inferences could also arise without key domain knowledge underpinning the interpretation of results.

## Conclusions

These scenarios and results illustrate the potential of ML algorithms to describe outbreak patterns, impute survival outcomes and thus improve CFR estimation. We confirm that adjusting for imperfect sensitivity and specificity reduces the bias in the CFR estimates based, in part, on imputed data. However, even with the adjustments, no method is robust enough to high percentages of missingness. Thus, a datacentric approach is recommended—patient survival outcome data collection should be prioritised in outbreak settings and random-sample follow-ups should be implemented to ascertain missing outcomes.

## Supporting information

S1 Appendix Supplementary information. Methodological details, additional results, and sensitivity analysis.
(DOCX)
