# Correction: Comparison of machine learning methods for estimating case fatality ratios: An Ebola outbreak simulation study

**DOI:** 10.1371/journal.pone.0316602

**Published:** 2024-12-26

**Authors:** The PLOS ONE Staff

The second affiliation of the first author is not indicated. Alpha Forna is also affiliated with #2: Department of Infectious Disease Epidemiology, MRC Centre for Global Infectious Disease Analysis, Imperial College London, London, United Kingdom.

There are errors in the Funding statement. The correct Funding statement is as follows: Dr Alpha Forna is funded by the Commonwealth Scholarship Commission, United Kingdom and a Simon Fraser University Postdoctoral Fellowship funded by Genome Canada. The UK Medical Research Council and Department for International Development (Centre funding)—grant number MR/R015600/1 supported all the authors: Prof Christl A Donnelly, Dr Pierre Nouvellet, Dr Ilaria Dorigatti and Dr Alpha Forna. The National Institute of Health Research (NIHR) supports Prof Christl A Donnelly through two grants: The Vaccine Efficacy Evaluation for Priority Emerging Diseases (VEEPED) [grant ref. NIHR: PR-OD-1017-20002] and the NIHR Health Protection Research Unit in Emerging and Zoonotic Infections [grant NIHR200907]. Dr Ilaria Dorigatti is funded by an Imperial College Junior Research Fellowship and a Sir Henry Dale Fellowship funded by the Royal Society and Wellcome Trust [grant 213494/Z/18/Z].

There is an error in reference 13. The correct reference is: WHO Ebola Response Team. Ebola virus disease in West Africa—the first 9 months of the epidemic and forward projections. N Engl J Med. 2014;2014(371):1481–95. pmid:25244186.

The publisher apologizes for the errors.
