# Supplementary material for: Comparison of machine learning methods for estimating case fatality ratios: An Ebola outbreak simulation study
Source: PLoS One. 2021 Sep 15;16(9):e0257005. doi: 10.1371/journal.pone.0257005 (PMC8443081; doi:10.1371/journal.pone.0257005)
Supplement: S1 Appendix — Methodological details, additional results, and sensitivity analysis. (DOCX) [file pone.0257005.s001.docx]

**S1 Appendix**

**Comparison of machine learning methods for estimating case fatality ratios: An Ebola outbreak simulation study.**

Alpha Forna^1^, PhD, Ilaria Dorigatti^2^, PhD, Pierre Nouvellet^2,3^ PhD, and Christl A. Donnelly^2,4^, ScD

1. School of Computing Science, Simon Fraser University, Burnaby, BC, V5A 1S6, Canada

2. MRC Centre for Global Infectious Disease Analysis, Department of Infectious Disease Epidemiology, Imperial College London, London, United Kingdom.

3. School of Life Sciences, University of Sussex, Brighton, UK.

4. Department of Statistics, University of Oxford, Oxford, UK.

**Corresponding author**: Alpha Forna

**Full Address**: Department of Computer Sciences, Burnaby Mountain Campus. 8888 University Drive Burnaby, B.C., Canada. V5A 1S6.

**Emai**l: [aforna@sfu.ca](mailto:aforna@sfu.ca)

Table of Contents

[Details of Method 3](#_Toc74723741)

[1.1 Generating Missingness 3](#_Toc74723742)

[1.1.1 Missing completely at random (MCAR) 3](#_Toc74723743)

[1.1.2 Missing at random (MAR) 3](#_Toc74723744)

[1.1.3 Missing not at random (MNAR) 3](#_Toc74723745)

[1.2 Algorithm implemented to investigate the performance of models as a function of outbreak data characteristics 3](#_Toc74723746)

[1.3 Algorithm implemented for CFR adjusted for bias in the models 5](#_Toc74723747)

[Additional results 6](#_Toc74723748)

[2.1 Survival outcome, demographic and clinical predictors considered for this simulation study 6](#_Toc74723749)

[2.2 Pairwise correlation between predictors 8](#_Toc74723750)

[Sensitivity analysis 9](#_Toc74723751)

[3.1 Comparing the unadjusted and adjusted CFR estimates for MNAR missingness in survival outcomes 9](#_Toc74723752)

# Details of Method

These algorithms follow closely those implemented in a preceding study (1). However, the algorithms were adapted to compare the five machine learning models (logistic regression [LR], random forest [RF], boosted regression trees [BRT], Bayesian additive regression trees [BART] and artificial neural network [ANN]) in a framework that ensured optimal hyperparameterisation of each model given the data.

## Generating Missingness

To investigate the impacts of the type of missingness, we simulated missingness in the survival outcome and the predictors of survival. The simulations for the different types of missingness are described as follows:

### 1.1.1 Missing completely at random (MCAR)

For *M%* data missingness, we drew a number from a uniform distribution *u* for every value in the dataset, and for values less than *M/100,* we replaced survival outcome with NA (i.e. the missing value). we used this same approach to generate MCAR missingness in the predictors.

### 1.1.2 Missing at random (MAR)

For *M*% data missingness, for every value of *u* greater than *M/100 (days),* we replaced survival outcome with NA (i.e. the missing value).

### 1.1.3 Missing not at random (MNAR)

For *M*% data missingness, I fitted a logistic regression using all predictors and survival outcome as the dependent variable, for every value of *u* greater than *M/100,* we replaced survival outcome with NA (i.e. the missing value). Thus, making the missingness depend on the survival outcome itself.

We used Little’s MCAR test (2) to differentiate between MCAR and non-MCAR missingness. We confirmed missingness was MAR by ensuring that we conditioned missingness in the survival outcome on reporting delay which is simulated as one of the observed predictors of outcome. We confirmed missingness was MNAR by ensuring that the missingness in survival outcome was conditional on the probability of the outcome itself.

The missingness in the predictors is simulated as MCAR in all scenarios investigated.

## Algorithm implemented to investigate the performance of models as a function of outbreak data characteristics

For given values of parameters characterising the simulated outbreak data; type of missingness in the survival outcome (MCAR, MAR, MNAR), proportion of outbreak data size (*p*), proportion of simulated missingness (*missingness*) and the proportion of cases in training data, LR, RF, BRT, BART and ANN models were built and validated using the following algorithm:

1. Generate the proportion of outbreak data (*p*) from the complete simulated data.
2. Estimate the true CFR for this simulated outbreak dataset
3. Generate the specific type of missingness (MCAR, MAR or MNAR) (as described in section 1.1) and proportion of missingness (*missingness*) of the survival outcome but generate MCAR missingness in the predictor each time.
4. Generate the training data by randomly sampling without replacement the specified proportions of cases with death and recovery outcomes.
5. Generate the out-of-sample validation data, consisting of the cases with observed survival outcomes excluded from the training data (step 4).
6. Build the LR, RF, BRT, BART and ANN models using functions in the ‘mlr’ package, using the training data built in step 1, the specified type of missingness in the survival outcome (MCAR, MAR, MNAR), proportion of outbreak data (*p*), proportion of simulated missingness (*missingness*) and the proportion of cases in training data, 5-fold cross validation and 50 random search iterations of the hyperparameters of each model using survival outcome (i.e. death or recovery) as response predictor and unexplained bleeding, fever, jaundice, fatigue, anorexia, vomiting, diarrhoea, headache, muscle pain, joint pain, chest pain, difficult breathing, conjunctivitis, confused, case classification, quarter, age, reporting delay, current hospitalisation status, country of origin as predictors. Optimal models minimising the holdout (cross validation) deviance are computed.
7. Use the optimal LR, RF, BRT, BART and ANN models built in step 6 to predict the survival outcomes of the cases in the validation data. For each subject i in the validation data, the model gives the probability $p_{i}$ that subject *i* is dead.
8. Define the cut-off threshold$p_{T}$ by which cases with$p_{i}$ > $p_{T}$ are classified as dead or alive otherwise. I chose cut-off values giving equal sensitivity and specificity using the ‘optimal.threshold’ function in the ‘PresenceAbsence’ package (3).
9. Use the cut-off threshold$p_{T}$ computed in step 8 to classify cases in the validation data as dead or alive.
10. Compute the sensitivity (proportion of deaths correctly classified), specificity (proportion of survival correctly classified) and percentage of percentage correctly classified in the validation data (PCC) and the area under the receiver operating characteristic curve (AUC).
11. Use the optimal models built in step 6 to calculate the survival probability of each case.
12. Use the cut-off threshold $p_{T}$ computed in step 8 to convert the survival probabilities to binary outcomes (i.e. death or recovery)
13. Calculate the CFR estimates and corresponding 95% confidence intervals using the cases with both imputed and observed survival outcomes.

The choice of optimising the cut-off thresholds to have equal sensitivity and specificity in step 5 was made to avoid introducing bias in CFR estimated with imputation.

## 1.3 Algorithm implemented for CFR adjusted for bias in the models

CFR estimates and confidence intervals adjusted for bias in the models (i.e. LR, RF, BRT, BART and ANN) were calculated from 100 realisations of steps 1 – 9, followed by steps 14-17 below:

1. Generate cases with just the imputed death outcomes.
2. Use the deaths in step 9, the total number of cases in the imputed data and *tp* function in the ‘RSurveillance’ package to estimate the CFR (4).
3. Multiply the number of cases in the imputed data by the inferred CFR to get the number of inferred deaths $f_{T}$.
4. The adjusted CFR is the ratio of the sum of $f_{T}$and the number of deaths in data with known survival outcomes, divided by the sum of all deaths and survivals in the complete data (i.e. data with both imputed and observed outcomes).

# Additional results

2.1 Survival outcome, demographic and clinical predictors considered for this simulation study.

Table 2.1 describes the survival outcome and predictors simulated in this study. Except for reporting delay (the difference between the date of reporting and the date of symptom onset in days) which is continuous, all other simulated predictors are categorical. Missingness in the survival outcome was simulated as MCAR/MAR/MNAR while missingness in all predictors was simulated as MCAR.

S1 Table: Survival outcome, demographic and clinical predictors considered for this simulation study.

| Predictor | Description | Mean (SD) | Type of missingness investigated |
| --- | --- | --- | --- |
| Outcome | | | |
| Survival outcome | Survival outcome in two classes: Alive, Dead | NA | MCAR/MAR/MNAR |
| Predictors | | | |
| Age (categorised) | Age in 16 classes: 0-4, 5-9,10-14,  15-19,20-24,  35-39,40-44,  45-49,50-54,  55-59,  60-64,65-69,  70-74,75+ | NA | MCAR |
| Country of origin | Country in three classes: Sierra Leone, Guinea, Liberia | NA | MCAR |
| Reporting delay | The difference between the date of reporting and the of symptom onset (days) | 5.68 (8.91) | MCAR |
| Case classification | Case classification in three classes: confirmed, probable and suspected | NA | MCAR |
| Hospitalisation status | Hospitalisation status in two classes: Yes, No | NA | MCAR |
| Quarter (Date of reporting aggregated at 3-month intervals) | Quarter in seven classes: January-March 2014, April-June 2014, July-September 2014, October-December 2014, January-March 2015, April-June 2015, July-September 2015 | NA | MCAR |
| Difficulty breathing | Difficult breathing in two classes: Yes, No | NA | MCAR |
| Fever | Fever in two classes: Yes, No | NA | MCAR |
| Fatigue | Fatigue in two classes: Yes, No | NA | MCAR |
| Anorexia | Anorexia in two classes: Yes, No | NA | MCAR |
| Unexplained bleeding | Unexplained bleeding in two classes: Yes, No | NA | MCAR |
| Confusion | Confusion in two classes: Yes, No | NA | MCAR |
| Joint pain | Joint pain in two classes: Yes, No | NA | MCAR |
| Jaundice | Jaundice in two classes: Yes, No | NA | MCAR |
| Conjunctivitis | Conjunctivitis in two classes: Yes, No | NA | MCAR |
| Vomiting | Vomiting in two classes: Yes, No | NA | MCAR |
| Diarrhoea | Diarrhoea in two classes: Yes, No | NA | MCAR |
| Headache | headache in two classes: Yes, No | NA | MCAR |
| Muscle pain | Muscle pain in two classes: Yes, No | NA | MCAR |
| Chest Pain | Chest pain in two classes: Yes, No | NA | MCAR |

*SD=standard deviation, * NA = Not applicable

## 2.2 Pairwise correlation between predictors


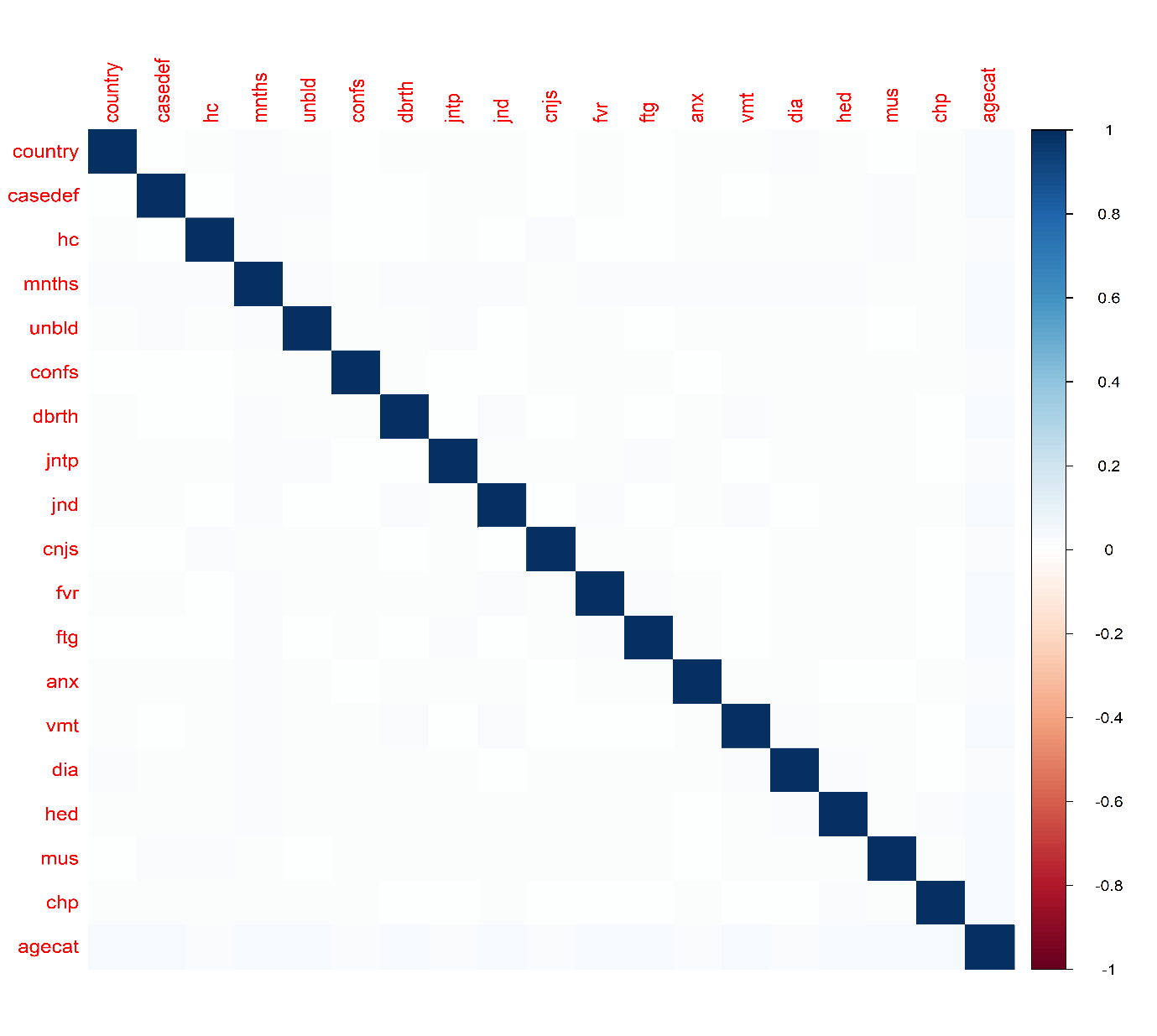


S1 Fig: Pairwise correlation between categorical predictors. Key: country= country of origin, casedef= case classification, hc = health care worker, mnths=quarter, unbld= unexplained bleeding, confs=confusion, dbrth= difficulty breathing, jntp= joint pain, jnd= jaundice, conjs= conjunctivitis, fvr= fever, ftg= fatigue, anx= anorexia, vmt= vomiting, dia= diarrhoea, hed= headache, mus= muscle pain, chp= chest pain, agecat= age as a categorical variable.

S2 Table: Pairwise Kruskal-Wallis p-values between the continuous predictor and other predictors.

The p-value >0.05 indicate weak correlation between predictors.

|  |  |
| --- | --- |
|  | Kruskal-Wallis p-values |
| delay | 0.178 |
| casedef | 0.512 |
| hc | 0.481 |
| mnths | 0.822 |
| unbld | 0.059 |
| confs | 0.484 |
| dbrth | 0.087 |
| jntp | 0.301 |
| jnd | 0.306 |
| cnjs | 0.841 |
| fvr | 0.344 |
| ftg | 0.993 |
| anx | 0.886 |
| vmt | 0.231 |
| dia | 0.296 |
| hed | 0.209 |
| mus | 0.529 |
| chp | 0.085 |
| agecat | 0.190 |

Key: country= country of origin, casedef= case classification, hc = health care worker, mnths=quarter, unbld= unexplained bleeding, confs=confusion, dbrth= difficulty breathing, jntp= joint pain, jnd= jaundice, conjs= conjunctivitis, fvr= fever, ftg= fatigue, anx= anorexia, vmt= vomiting, dia= diarrhoea, hed= headache, mus= muscle pain, chp= chest pain, agecat= age as a categorical variable.

# Sensitivity analysis

As a sensitivity analysis, we present results for the comparison of unadjusted and adjusted CFR for MNAR missingness in the survival outcomes.

## 3.1 Comparing the unadjusted and adjusted CFR estimates for MNAR missingness in survival outcomes

S2 Fig shows that for MNAR missingness in survival outcome and after sensitivity and specificity adjustment, overall reduction in CFR bias across inference methods, proportion of missingness, outbreak size and proportion of training data was 1.5% (median, range: 0%–16%).


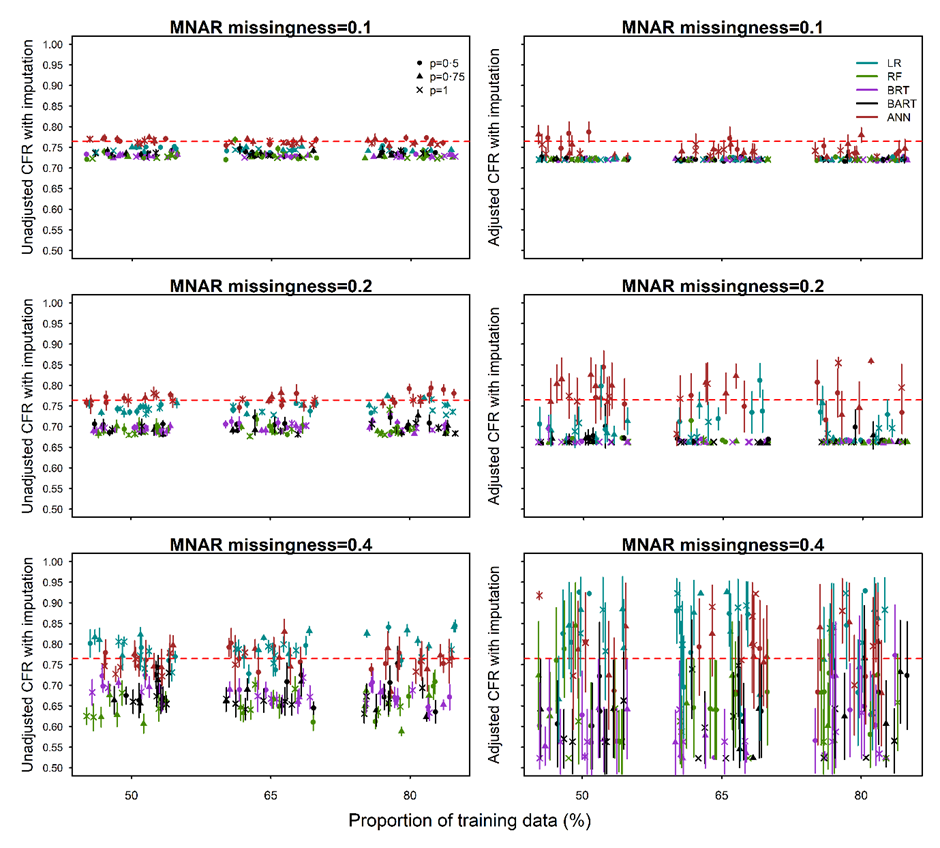


S2 Fig: Comparing the unadjusted and adjusted CFR estimates for MNAR missingness in survival outcomes and predictors missing completely at random (MCAR). The true CFR of the complete simulated data (without simulated missingness) is indicated by the red dotted horizontal line.

**References**

1. Forna A, Nouvellet P, Dorigatti I, Donnelly CA. Case Fatality Ratio Estimates for the 2013–2016 West African Ebola Epidemic: Application of Boosted Regression Trees for Imputation. Clinical Infectious Diseases. 2019;70(12):2476-83.

2. Li C. Little's test of missing completely at random. The Stata Journal. 2013;13(4):795-809.

3. Freeman ME. Package ‘ PresenceAbsence ’. 2015.

4. Sergeant E. Package ‘ RSurveillance ’. 2016.
